# The Mrs1 Splicing Factor Binds the bI3 Group I Intron at Each of Two Tetraloop-Receptor Motifs

**DOI:** 10.1371/journal.pone.0008983

**Published:** 2010-02-01

**Authors:** Caia D. S. Duncan, Kevin M. Weeks

**Affiliations:** Department of Chemistry, University of North Carolina, Chapel Hill, North Carolina, United States of America; Lehigh University, United States of America

## Abstract

Most large ribozymes require protein cofactors in order to function efficiently. The yeast mitochondrial bI3 group I intron requires two proteins for efficient splicing, Mrs1 and the bI3 maturase. Mrs1 has evolved from DNA junction resolvases to function as an RNA cofactor for at least two group I introns; however, the RNA binding site and the mechanism by which Mrs1 facilitates splicing were unknown. Here we use high-throughput RNA structure analysis to show that Mrs1 binds a ubiquitous RNA tertiary structure motif, the GNRA tetraloop-receptor interaction, at two sites in the bI3 RNA. Mrs1 also interacts at similar tetraloop-receptor elements, as well as other structures, in the self-folding *Azoarcus* group I intron and in the RNase P enzyme. Thus, Mrs1 recognizes general features found in the tetraloop-receptor motif. Identification of the two Mrs1 binding sites now makes it possible to create a model of the complete six-component bI3 ribonucleoprotein. All protein cofactors bind at the periphery of the RNA such that every long-range RNA tertiary interaction is stabilized by protein binding, involving either Mrs1 or the bI3 maturase. This work emphasizes the strong evolutionary pressure to bolster RNA tertiary structure with RNA-binding interactions as seen in the ribosome, spliceosome, and other large RNA machines.

## Introduction

RNA and proteins have co-evolved to form the ribonucleoproteins (RNPs) that now carry out many of the fundamental steps of gene regulation, including mRNA processing and protein biogenesis [1]. Core functions of complexes such as the spliceosome and the ribosome are performed in active sites composed of RNA; however, these RNA elements also require extensive participation by protein facilitators [1]. Similarly, most group I introns likely require protein cofactors to catalyze their own excision from flanking exons and to splice efficiently. Group I introns, therefore, represent ideal models for testing the role of protein recruitment into ribonucleoprotein complexes.

The group I intron active site is composed of RNA. The catalytic core is formed at the interface of three RNA domains, termed the P1-P2, the P5-P4-P6, and the P9-P7-P3-P8 domains. These domains are held in a precise and catalytically active three-dimensional architecture by inter-domain tertiary interactions [2], [3], [4], [5]. In a few minimal group I introns, these tertiary interactions involve direct and compact interactions between RNA domains. However, most group I introns are more complex. In general, group I introns have evolved large peripheral RNA elements and have recruited a wide range of protein cofactors to stabilize their active conformations [2], [6], [7], [8], [9], [10].

Protein cofactors use diverse strategies to stabilize group I intron RNA tertiary structure. Proteins such as CYT-18 bind multiple RNAs by recognizing conserved elements in the group I intron catalytic core [11]. Alternatively, proteins including CBP2 [12], [13] and maturase proteins [14], [15] recognize specific introns through interactions with idiosyncratic peripheral elements. Many group I intron splicing factors have been co-opted or evolved from proteins that perform other nucleic acid binding functions. In the cases of the maturase proteins and Pet54, an existing nucleic acid binding surface is reused to accommodate the new group I intron substrate [14], [16]. Alternately, CYT-18 has evolved separate binding surfaces to perform distinct functions as a group I intron cofactor and as a tRNA synthetase [11], [17].

The yeast mitochondrial bI3 group I intron is an instructive example of an RNA that has become dependent on proteins to fold and function correctly. bI3 RNA splicing requires specific binding by two proteins, the bI3 maturase and two dimers of the Mrs1 protein [18], [19]. The free RNA is extensively misfolded and binding by the maturase and Mrs1 proteins induces large conformational rearrangements in both secondary and tertiary structure [20]. The bI3 maturase protein binds to the P5-P4-P6 domain and promotes formation of long-range tertiary interactions to stabilize the P5 and P4 components of the catalytic core [14]. The Mrs1 protein facilitates splicing for both the bI3 and aI5β introns in yeast mitochondria [21], even though these two introns are not especially similar [2]. Mrs1 is related to the RuvC family of DNA junction resolvases and, in evolutionary terms, appears to have acquired an RNA binding activity only recently and in a small subset of organisms [22]. Mrs1 may have retained its nucleic acid binding site but is no longer capable of cleaving DNA [18], [22]. At present, the RNA binding site and molecular function of Mrs1 in group I intron splicing are unexplored.

In this work, we use high-throughput hydroxyl radical footprinting to identify the RNA binding sites for Mrs1. Mrs1 binds at each of two conserved GNRA tetraloop-receptor interactions in the bI3 RNA (where N is any loop nucleotide and R is a purine). The tetraloop-receptor motif was one of the first long-range tertiary interactions to be identified for RNA [23]. This interaction involves hydrogen bonding between GNRA loop nucleotides with functional groups in the minor groove of the receptor helix [23], [24], [25]. We also show that Mrs1 interacts at tetraloop-receptor elements in the *Azoarcus* group I intron and the *Bacillus subtilis* ribonuclease P (RNase P) specificity domain RNA and at other sites in these non-cognate RNAs. These data indicate that Mrs1 has a general affinity for RNA coupled with selectivity for the GNRA tetraloop-receptor interaction, especially in its cognate bI3 RNA. Thus, Mrs1 has evolved from a DNA-binding resolvase to a protein capable of binding the GNRA tetraloop-receptor motifs found ubiquitously in large RNAs. This work also emphasizes the strong evolutionary pressure to usurp RNA-only structures with RNA-protein interactions. Every long-range RNA tertiary interaction in the bI3 ribonucleoprotein is stabilized by either the maturase or Mrs1 proteins and both proteins have been co-opted from earlier DNA-binding functions.

## Results

### Mrs1 Binds and Stabilizes Tetraloop-Receptor Interactions in the bI3 RNA

**Figure 1:**
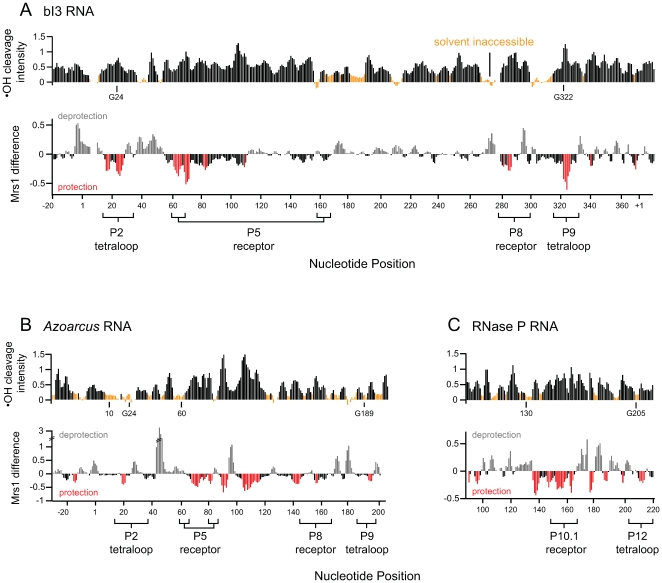
High-throughput hydroxyl radical footprinting analysis of Mrs1 binding to RNA. (Top panels) Histograms of cleavage intensity versus nucleotide position for the free (A) bI3 group I intron, (B) *Azoarcus* group I intron, and (C) RNase P specificity domain. Solvent inaccessible nucleotides in the free RNA (intensities ≤ one-half the mean) are orange. (Bottom panels) Difference plots reporting cleavage intensities for the Mrs1-bound RNA minus the free RNA. Significant cleavage protections are emphasized in red. The first guanosine residue in each GNRA tetraloop is labeled explicitly. Bars below the axes indicate tetraloop and receptor helix structure landmarks.

We identified RNA interaction sites for the Mrs1 protein using hydroxyl radical footprinting. Hydroxyl radicals are generated *in situ* from H_2_O_2_ in the presence of a Fe(II)-EDTA catalyst [26], [27]. The hydroxyl radicals then cleave the RNA backbone in a way that is roughly correlated with solvent accessibility [28], [29]. We performed high-throughput hydroxyl radical experiments by identifying cleavage positions using primer extension performed with fluorescently labeled primers, resolved by capillary electrophoresis [30], [31], [32]. This experiment yields single nucleotide resolution cleavage information for the entire 540 nucleotide bI3 splicing precursor in one high-throughput experiment (top panel, Figure 1A). Cleavage intensities were normalized to a scale from 0 to ∼1.5, where 1.0 is defined as the average intensity of highly reactive nucleotides. On this scale, we classify nucleotides with reactivities that are one-half the mean or less as solvent inaccessible (Figure 1A, orange columns).

In the free bI3 RNA, only ∼20% of nucleotides are protected from cleavage prior to binding by the Mrs1 protein (top panel, Figure 1A). Most RNA elements expected to form tertiary contacts are reactive, including the entire P5-P4-P6 domain, the GNRA tetraloops at the ends of the P2 and P9 helices (G of each tetraloop is labeled in Figure 1A), and their respective receptors in the P8 and P5 helices (labeled, Figure 1A). These results indicate that, prior to protein binding, the bI3 RNA contains some tertiary structure but, overall, is not folded in a catalytically active structure.

Upon addition of Mrs1, extensive regions in the RNA became protected from cleavage. We quantified the effect of Mrs1 binding using a difference plot in which the hydroxyl radical cleavage intensities for the free RNA were subtracted from those for the Mrs1-bound RNA (lower panel, Figure 1A). Because the results of hydroxyl radical cleavage experiments are highly quantitative when resolved by capillary electrophoresis, difference plots represent a simple model-free approach for visualizing Mrs1-induced changes in RNA structure. We observe both significant protections and deprotections upon Mrs1 binding. Protections and enhancements are reported as negative and positive differences, respectively (in red and gray, Figure 1). We define significant changes as those corresponding to an absolute reactivity difference of 0.2 or greater (which is 2-fold above the mean background). Almost all significant protections occur in or are immediately adjacent to RNA structures that participate in one of two tetraloop-receptor interactions in the bI3 RNA (in red, Figure 1A). These tetraloop-receptor interactions link the L2 loop to the P8 helix and the L9 loop to the P5 helix. Combined, these two tertiary interactions structurally link the three group I intron RNA domains. The observed protections include both regions expected to reflect physical contacts between the tetraloop and receptor helix structures and also RNA regions facing the exterior of the RNA that report Mrs1-RNA interactions. In addition, Mrs1 binding caused a few RNA elements to become more reactive, consistent with protein-induced conformational changes (in gray; lower panel, Figure 1A). These results provide strong evidence that Mrs1 binds to and stabilizes both GNRA tetraloop-receptor interactions in the otherwise misfolded bI3 RNA.

### Mrs1 Interacts at Tetraloop-Receptor Motifs in Non-Cognate RNAs

**Figure 2:**
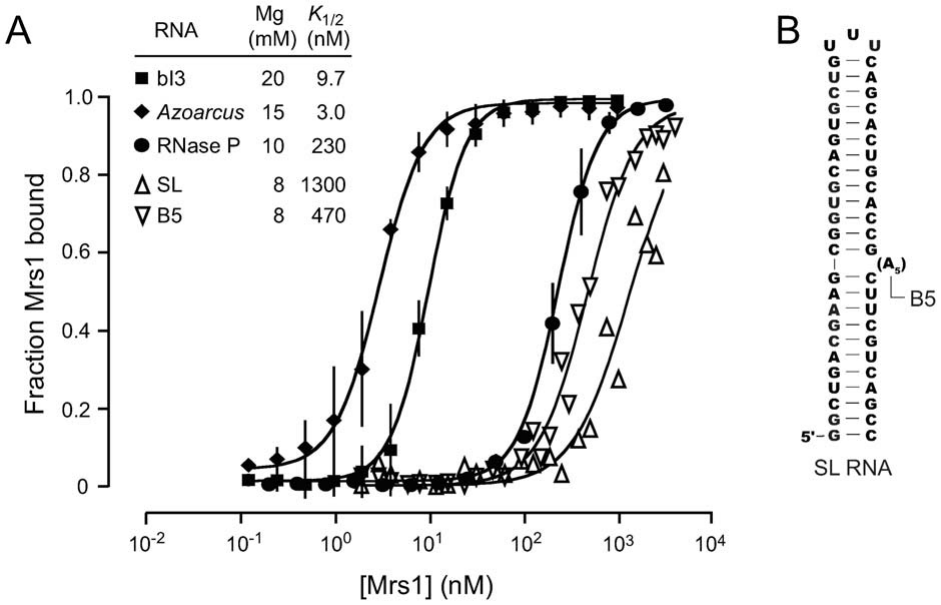
Mrs1 binding to the bI3, *Azoarcus*, RNase P, and simple stem-loop RNAs. (A) Binding experiments were performed in duplicate under conditions optimal for each RNA [18], [39], [40]. Both *K*_1/2_ and the Hill coefficient were fit independently for each RNA, *R* ^2^ values were ≥0.9 in all cases. (B) Structures of the stem-loop (SL) and five nucleotide bulge (B5) RNAs.

We tested whether Mrs1 might generally be able to bind tetraloop-receptor motifs by evaluating binding to two other RNAs that contain this interaction. The *Azoarcus* group I intron and RNase P specificity domain RNAs contain two and one GAAA tetraloop-receptor motif, respectively. In contrast to the bI3 RNA, these RNAs are relatively small and contain well-characterized tetraloop-receptor elements that fold independently and accurately in the presence of magnesium ions [25], [33]. The tetraloop-receptor motifs in the *Azoarcus* and RNase P RNAs also contain slightly more elaborate receptor helices, which likely contribute to their stability as independent RNA elements [23]. We evaluated Mrs1 binding to all three RNAs under conditions that support accurate folding for each RNA using filter partitioning experiments (solid symbols, Figure 2A). Consistent with previous work [18], two Mrs1 dimers cooperatively bind the bI3 RNA with a Hill coefficient of 2.2 and a *K*_½_ of 9.7 nM (square symbols, Figure 2A). The *Azoarcus* group I intron binds Mrs1 more tightly than does the cognate bI3 RNA: the *K*_½_ is 3.0 nM with a Hill coefficient of 1.8. RNase P binds significantly more weakly, the *K*_½_ is 230 nM with a Hill coefficient of 2.0.

The Hill coefficient gives a measure of the minimum numbers of binding events. Protein binding to all three RNAs is suggestive of cooperative binding by at least two Mrs1 dimers (Figure 2A). Cooperative binding to the *Azoarcus* intron is consistent with the presence of two tetraloop-receptor interactions in this RNA, as is also the case for the bI3 RNA. In contrast, the RNase P RNA contains a single tetraloop-receptor motif. The apparent cooperativity may reflect a second, weaker, Mrs1 binding site as reflected by the larger *K*_½_ for RNase P as compared to the two group I intron RNAs.

High-throughput hydroxyl radical experiments indicate that the free *Azoarcus* and RNase P RNAs fold into their active conformations. For example, both *Azoarcus* and RNase P RNAs are protected from hydroxyl radical cleavage in the elements that comprise the tetraloop-receptor interactions in these RNAs (tetraloop and receptor sequences are identified below the axis and the position of the first G in each tetraloop is labeled in Figures 1B,C). In addition, other elements that form key tertiary interactions are also protected from hydroxyl radical cleavage in these RNAs. These protections include the docking interaction of the *Azoarcus* P1 helix (at position 10) with the P4 helix (position 60) and the stacking of RNase P nucleotide A130 in the P11 helix (labeled in upper panels, Figures 1B,C).

**Figure 3:**
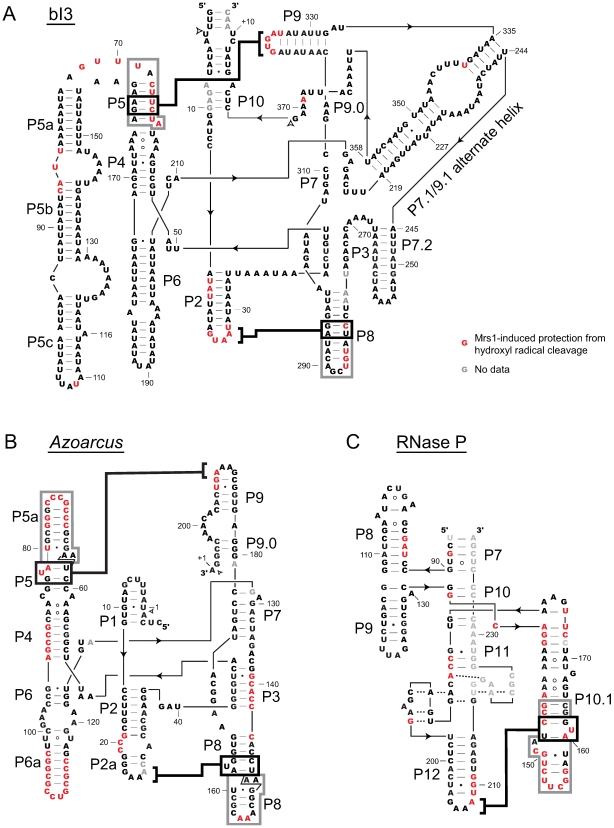
Secondary structure models illustrating Mrs1 protections for the bI3, *Azoarcus*, and RNase P RNAs. Nucleotides protected from hydroxyl radical cleavage upon Mrs1 binding are red. Bold black lines emphasize tetraloop-receptor interactions; protected residues adjacent to the receptor helices are emphasized with gray boxes. Paired helical regions are indicated by P*x*. Group I intron splice sites are indicated with open arrows. A small number of sites with no data, due to high background, are shown in gray lettering.

We visualized protections from hydroxyl radical cleavage, induced upon Mrs1 binding, again using quantitative difference plots (red columns, Figures 1B,C). Even though these RNAs are highly structured prior to protein binding, Mrs1 protects all three tetraloop-receptor motifs in the *Azoarcus* and RNase P RNAs from hydroxyl cleavage. The loop elements are P2 and P9 in the *Azoarcus* RNA and P12 in RNase P. The receptor helices are P5 and P8 in the *Azoarcus* RNA and P10.1 in the RNase P RNA (see heavy black and gray lines, Figures 3B,C). Binding by Mrs1 additionally protects other regions, not involving the tetraloop-receptor motif, from cleavage in both the *Azoarcus* and RNase P RNAs. These extra protections fall primarily in the *Azoarcus* P6a helix and in the RNase P central junction and adjoining regions of the P10.1 helix (Figures 3B,C). These observations suggest that Mrs1 shows good, but imperfect, specificity for the tetraloop-receptor motif in these RNAs.

We therefore assessed the general ability of Mrs1 to bind simple RNA motifs by evaluating binding to two additional RNAs, an RNA stem-loop containing 24 continuous base pairs and a similar stem-loop RNA containing a five nucleotide bulge, termed the SL and B5 RNAs, respectively (Figure 2B). Mrs1 binds to the SL RNA weakly, with a *K*_1/2_ of 1.3 µM; binding to the B5 RNA has a slightly higher affinity of ∼500 nM (open symbols, Figure 2A). In both cases, the Hill coefficient is ∼1.5. These experiments were conducted at 8 mM MgCl_2_ concentrations because binding was undetectable at 15–20 mM divalent ion. Together, the binding and hydroxyl radical footprinting data indicate that Mrs1 binds weakly at irregular structures in RNA, which may reflect its recent evolution from a DNA junction-binding protein [18], and shows a preference for interacting at the tetraloop-receptor motif, if present.

The five tetraloop-receptor interactions present in the three RNAs are in different local structural contexts and have different extents of preexisting structure, prior to Mrs1 binding. Despite these differences, the net pattern of protection from hydroxyl radical cleavage upon Mrs1 binding is similar. Protected regions include (*i*) the tetraloop itself, (*ii*) nucleotides in the receptor helix where the tetraloop interacts, and (*iii*) a region in the receptor helix extending towards the exterior of each RNA (emphasized with black and gray boxes, Figure 3). Mrs1 thus appears to interact with both the tetraloop and the receptor helix elements of this motif.

## Discussion

The Mrs1 protein binds to and promotes the formation of two distinct GNRA tetraloop-receptor interactions in the bI3 group I intron RNA (Figure 3A). Mrs1 also binds structurally homologous, but non-cognate, tetraloop-receptor interactions in the *Azoarcus* group I intron and RNase P specificity domain RNAs. In addition, Mrs1 binds other sites in the non-cognate *Azoarcus* and RNase P RNAs and, with lower affinity, to a bulged stem-loop motif. Thus, Mrs1 appears to have the general ability to bind irregular RNA structures with a preference for interacting at the tetraloop-receptor motif.

Mrs1 binding is also required for splicing of a second group I intron, the aI5β intron from the COX1 pre-mRNA in yeast mitochondria [21]. Similar to the bI3 RNP, two Mrs1 dimers bind the (1574 nt) aI5β RNA; however, in contrast to the bI3 RNA, binding is not reported to be cooperative [34]. The aI5β intron contains the L9-P5 tetraloop-receptor interaction but lacks the second P2-P8 interaction found in the bI3 RNA, which may explain the absence of cooperative binding. Given its large size, other tetraloop-receptor motifs or secondary binding sites for Mrs1 may exist in the aI5β intron.

**Figure 4:**
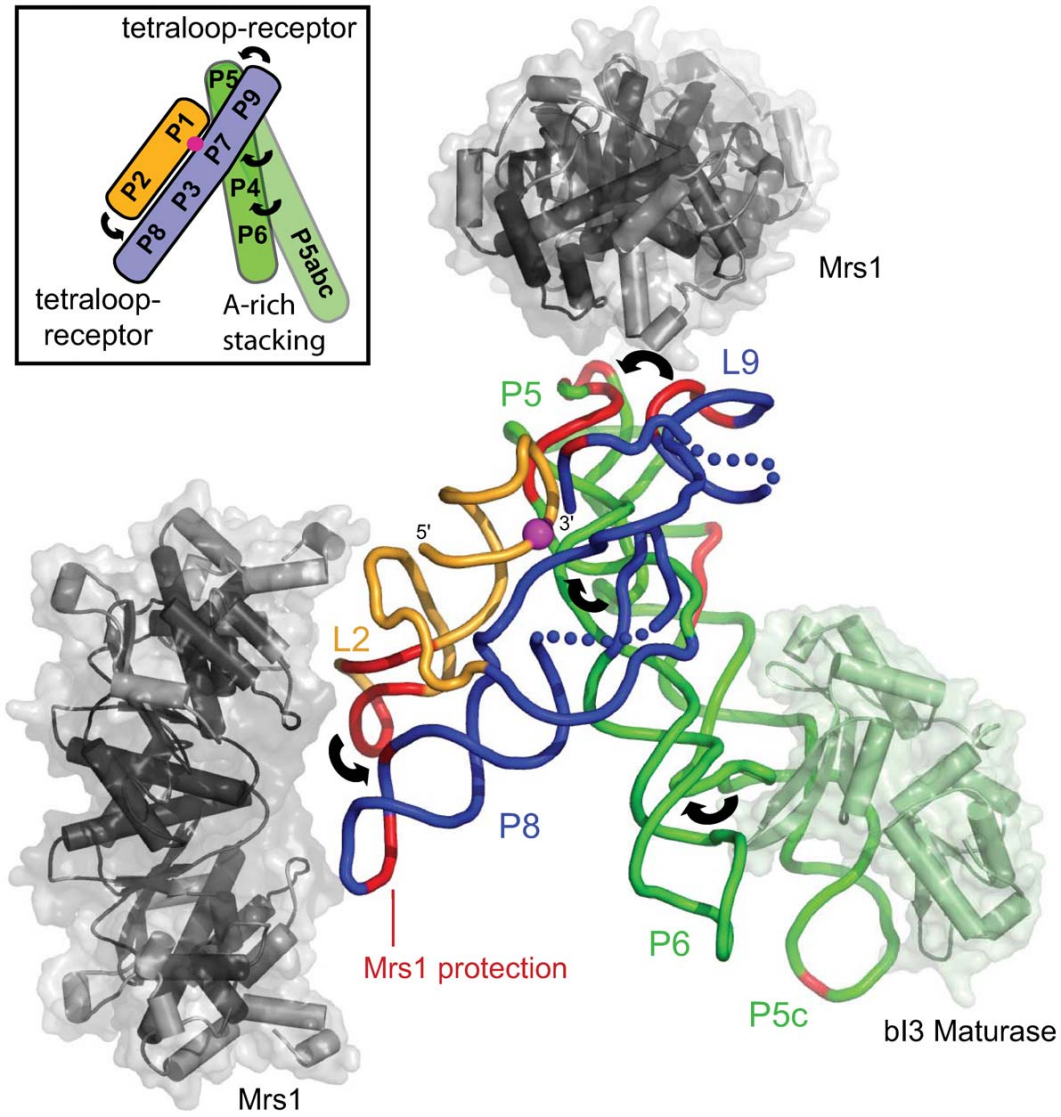
Structural model for the six-component bI3 ribonucleoprotein complex. The RNA backbone is shown as a tube and is colored by domain. Long-range tertiary interactions are emphasized with curved arrows in both the main figure and in the inset. Backbone positions protected by Mrs1 binding are red. Mrs1 dimers are colored dark and light gray in regions of high and moderate structural homology, respectively, relative to its Ydc2 homolog. The bI3 maturase [14] is light green. The scissile phosphate at the active site is shown with a magenta sphere.

Identification of two tetraloop-receptor interactions as the Mrs1 binding sites in the bI3 RNA both rationalizes why two dimers of Mrs1 are required for full folding and catalytic activity in the intron [18] and also provides a basis for understanding the global architecture of the six-component bI3 RNP. We developed a three-dimensional model for the bI3 intron RNA in its catalytically active conformation by grafting the bI3 sequence onto known group I intron structures [12], [17], [25], [35], [36] (Figure 4). The structure of the bI3 maturase protein was previously solved by crystallography and shown to bind to the peripheral P5c helix [14] (in green, Figure 4).

We generated a model for Mrs1 by threading [37] the Mrs1 sequence against its nearest homologue, Ydc2 [38]. Strong structural homology is apparent for the α-helices at the dimer interface and for the central β-sheet in each monomer (emphasized in dark gray, Figure 4). Residues whose positions are less well established by threading lie on the periphery of the protein (in light gray, Figure 4). Modeling supports the view that, like its homologues, Mrs1 is an extended dimer with an axial ratio of ∼2∶1. The nucleic acid binding site is located on one side of the long face.

Nucleotides protected by Mrs1 occur predominantly at the tetraloop-receptor interactions involving L2-P8 and L9-P5 (in red, Figure 4). We assume Mrs1 uses approximately the same nucleic acid binding cleft for RNA as its homologues do for DNA. The tetraloop-receptor RNA elements fit best in the Mrs1 binding site when positioned parallel to the long axis of the protein. The two tetraloop-receptor motifs in the bI3 RNA are related by a ∼90° rotation; thus, the long axes of the two Mrs1 dimers are roughly perpendicular (Figure 4).

The catalytic active site of the intron is located roughly at the center of the RNP complex (the scissile phosphate cleaved in the first step of splicing is shown as a magenta sphere, Figure 4). This active site is stabilized by three long-range tertiary interactions located 30–50 Å from the scissile phosphate (illustrated schematically with curved arrows, Figure 4). All three bound proteins share critical features: (*i*) each protein stabilizes a crucial long-range RNA tertiary interaction, (*ii*) no protein binds within ∼20 Å of the scissile phosphate and (*iii*) no protein binds within ∼50 Å of another protein.

The six-component bI3 complex represents a catalytic RNA caught in the act of becoming an obligate ribonucleoprotein. The catalytic active site is still composed of RNA and no protein appears to approach closer than ∼20 Å to the scissile phosphate. However, in the bI3 RNP, every long-range RNA interaction is stabilized by a bound protein cofactor. Moreover, each protein has been co-opted from a prior DNA-binding function, suggesting that evolution to an RNA splicing factor is a recent event in evolutionary terms. Similar to many proteins in larger RNPs, these essential proteins bind distally from the catalytic core and induce significant effects on RNA structures up to 50 Å away. This work thus provides a clear example of the strong evolutionary pressure to recruit protein cofactors to facilitate formation of RNA tertiary structure for RNA-centered reactions in biology.

## Materials and Methods

### RNA Constructs and Protein Expression

The bI3 intron (with flanking exon sequences of 84 and 90 nucleotides) and the RNase P specificity domain (with flanking structure cassette sequences) RNAs were generated *in vitro* as described [20], [39]. The *Azoarcus* RNA included the tRNA exon [40] and 5′ and 3′ structure cassette sequences [41] and was generated by *in vitro* transcription [1 mL, 25°C, 6 h; containing 40 mM Tris (pH 7.5), 5 mM MgCl_2_, 2 mM spermidine, 10 mM DTT, 0.001% (v/v) Triton X-100, 0.166 µg of pyrophosphatase (Roche), 2 mM each nucleotide triphosphate, ∼10 µg double stranded DNA PCR template, 60 units SUPERNase-In (Ambion), 70 µg T7 polymerase], and purified by gel electrophoresis. The SL and B5 RNAs were synthesized from single stranded DNA templates with a double stranded promoter region. Mrs1 was expressed and purified as described [18] except that Mops was used as the buffer, the concentration of DTT was 5 mM, and glycerol was 10% (v/v).

### High-Throughput Hydroxyl Radical Cleavage Experiments and Analysis

Each RNA (3 pmol) was renatured under conditions previously shown to be optimal for folding of the specific RNA. The bI3 RNA was incubated in water at 95°C for 1 min, 4°C for 1 min, and 37°C for 10 min in reaction buffer [40 mM Hepes (pH 8.0), 80 mM potassium acetate (pH 8.0), 20 mM MgCl_2_] [18]; the *Azoarcus* RNA was incubated [in 25 mM Hepes (pH 7.5), 15 mM MgCl_2_] at 52°C for 5 min, and 37°C for 2 min [40]; and the RNase P RNA was incubated in water at 95°C for 2 min, 4°C for 2 min, and 37°C for 20 min [in 100 mM Mops (pH 8.0), 100 mM NaCl, 10 mM MgCl_2_] [39]. Mrs1 dimers were incubated with each RNA at 5-fold molar excess (0.5 µM final concentration), for 30 min at 37°C. Cleavage reactions (30 µl final volume; 5 min at 37°C) were initiated by the sequential addition of 3 µl each of 5.0 mM (NH_4_)Fe(SO_4_)_2_–7.5 mM EDTA (pH 8.0), 0.1% H_2_O_2_, and 50 mM sodium ascorbate [bI3 reactions were performed at one-half this Fe(II)-EDTA concentration]. Background was assessed using a reaction omitting Fe(II)-EDTA. Reactions were quenched with glycerol to a final concentration of 30% (v/v). Mrs1 was removed by proteinase K digestion [60 µg, 37°C, 30 min] followed by phenol:chloroform:isoamyl alcohol (25∶24∶1) extraction. Reactions were recovered by precipitation with ethanol. Sites of cleavage were identified by primer extension using fluorescently labeled primers [6-FAM, (+) Fe(II)-EDTA; HEX, (–) Fe(II)-EDTA; NED, ddT sequence ladder)] in 50 mM Tris-HCl (pH 8.3), 75 mM KCl, 3 mM MgCl_2_, 5 mM DTT, 0.5 mM each dNTP, and 100 units of Superscript III reverse transcriptase (Invitrogen). Primer extension reactions corresponding to the plus and minus Fe(II)-EDTA and sequencing lanes were combined, precipitated with ethanol, dissolved in formamide and separated on an Applied Biosystems 3130 capillary electrophoresis instrument. Fluorescent cleavage data were analyzed using ShapeFinder [20], [31]. Integrated intensities were normalized by dividing the data set by the average of the 8% most reactive nucleotides after first excluding the top 2% of reactivities. By this definition, 1.0 is the mean cleavage intensity of the most highly reactive nucleotides. Cleavage intensities were smoothed over a three nucleotide window for visualization.

### Mrs1 Binding Assays

Mrs1-RNA binding assays were performed by filter partitioning using preincubated nitrocellulose (Whatman) and Hybond (Amersham) filters [19]. Either [^32^P] 5′-end-labeled (∼0.5 nM, large RNAs) or internally labeled (SL and B5, ∼0.1 nM, using the bI3 conditions) RNAs were folded in their respective buffers and incubated with Mrs1 for 30 min at 37°C before filtering. Wells were washed with 3 vol of reaction buffer before and after binding. Filters were quantified by phosphorimaging (Molecular Dynamics) and fit to an equation that accounts for cooperative binding by two dimers of Mrs1 [18], fraction RNA bound  =  *A*([P]*^n^* / [P]*^n^* + *K*_1/2_ *^n^*), where *A* is the total fraction of RNA bound (typically ≥0.95), [P] is the protein concentration, *n* is the apparent Hill coefficient, and *K*_½_ is the concentration of Mrs1 where one-half the RNA is bound. In all cases, RNA concentrations were at least 5-fold below the measured *K*_1/2_ values to ensure validity of the binding equation. For all binding curves, *R* ^2^ was ≥0.9.

### Mrs1 and bI3 RNA Models

The Mrs1 protein model was generated with I-TASSER [37], [42] using Ydc2 [38] as the template structure. Core Mrs1 protein structural elements had RMSD values that differed by less than 3.4 Å between the five output models and less than 5.5 Å when compared to Ydc2 (calculated using lsqman [43]). The center-most model, with the smallest RMSD compared to the other models, was chosen as the representative for Figure 4. A model for the bI3 RNA was assembled from crystal structures [35], [36] using lsqman and Sybyl (Tripos). The structure was further refined by discrete molecular dynamics [44] adding base pairing, harmonic constraints around core elements, and constraints for long-range tertiary interactions. Structure images were composed with Pymol (Delano Scientific).
